# Room-Temperature Aqueous Synthesis of Amino-Functionalized Al-Based MOF as a Ratiometric Fluorescent Probe for Aqueous Dichromate Detection

**DOI:** 10.3390/molecules31173051

**Published:** 2026-08-31

**Authors:** Chuyao Huang, Yanxiu Zhang, Shu Li, Rui Lin, Yunfan Zhang, Jingqi Chen, Yutong Sun, Yue Wang, Shuo Liu

**Affiliations:** 1 School of Energy and Chemical Engineering, Tianjin Renai College, Tianjin 301636, China; huangchuyaohcy@163.com (C.H.); 15390648526@163.com (Y.Z.);; 2 Technology Center of Shenyang Customs, Shenyang 110016, China; linda_0915@126.com

**Keywords:** metal–organic framework, ratiometric fluorescence, dichromate anion, fluorescent sensing

## Abstract

In this work, amino-functionalized Al-based MOF Al-GM was fabricated via a mild room-temperature aqueous route, which was applied as a ratiometric fluorescent probe for the specific detection of Cr_2_O_7_^2−^ in water. Characterizations including XRD, FT-IR, SEM, and BET verify that Al-GM synthesized in pure water exhibits high crystallinity and abundant mesoporous channels, with fully exposed amino recognition sites on the framework, delivering dual fluorescence emission signals. Sensing performance experiments demonstrate outstanding selectivity toward Cr_2_O_7_^2−^ with negligible interference from coexisting metal ions. Fluorescence titration reveals a wide linear detection range and an ultralow limit of detection, which is far lower than the discharge standard of Cr(VI) for industrial wastewater. pH-dependent tests confirm the stable sensing performance of the probe in water at a pH range of 5–9. XRD and FTIR spectra before and after Cr_2_O_7_^2−^ adsorption confirm intact crystal and organic coordination frameworks during ion recognition. Cr_2_O_7_^2−^ anions are selectively captured by the synergy of electrostatic attraction and intermolecular hydrogen bonds with amino sites. This study proposes a mild, organic-solvent-free synthetic strategy for MOFs, and the prepared Al-GM displays promising application potential for trace Cr(VI) monitoring in aquatic environments.

## 1. Introduction

With the rapid development of industry and agriculture, heavy metal pollution has become one of the most serious environmental threats worldwide [1,2,3]. As a typical toxic heavy metal anion, dichromate (Cr_2_O_7_^2−^) is widely used in electroplating, metallurgy, printing, and dyeing industries. However, Cr_2_O_7_^2−^ exhibits high water solubility, strong mobility, and severe biological toxicity, which can cause irreversible damage to the liver, kidney, digestive system, and even induce gene mutation and cancer. Excessive Cr_2_O_7_^2−^ discharged into water bodies seriously destroys aquatic ecological balance and eventually endangers human health through the food chain [4,5,6]. Therefore, the development of a rapid, sensitive, and reliable method for the detection of Cr_2_O_7_^2−^ in environmental water samples is of great practical significance.

Among various detection techniques, fluorescence sensing has attracted extensive attention owing to its simple operation, fast response, high sensitivity, low cost, and potential for on-site visual detection. Compared with single-emission fluorescent probes susceptible to matrix interference and instrumental drift, ratiometric fluorescent sensors employ two independent luminescence signals for built-in self-calibration, which effectively suppress extrinsic disturbances and improve the reliability of quantitative analysis [7,8,9]. Thus, the design and construction of high-performance ratiometric fluorescent probes for the efficient recognition of Cr_2_O_7_^2−^ have become a research hotspot in the field of environmental analysis [10,11].

Metal–organic frameworks (MOFs), as a class of crystalline porous materials assembled by metal nodes and organic ligands, have been widely used for fluorescence-sensing, adsorption, separation, and catalysis due to their large specific surface area, adjustable pore structure, abundant active sites, and designable luminescence properties [12,13,14,15]. Luminescent MOFs can achieve specific recognition and signal response toward target analytes through host–guest interactions, energy transfer, electron transfer, or competitive absorption, showing unique advantages in the detection of heavy metal ions and oxysalts [16,17,18]. As an important subclass of porous MOF materials, the MIL-53 family possesses prominent thermal and chemical stability derived from one-dimensional metal–oxygen chains [19]. Among them, amino-functionalized NH_2_-MIL-53(Al) has drawn wide attention because abundant amino groups on organic ligands can serve as recognition sites for target analytes [20,21,22]. Nevertheless, conventional preparation of NH_2_-MIL-53(Al) mainly depends on solvothermal approaches that require high-temperature autoclave conditions and a toxic DMF solvent, accompanied by high energy consumption and complicated post-treatment procedures, which severely restrict its large-scale practical application and conflict with green-chemistry principles [23,24,25,26].

To overcome the shortcomings of solvothermal routes, ambient-temperature aqueous synthesis of NH_2_-MIL-53(Al) has been reported in previous literature [27,28,29]. Among them, one protocol introduced an additional CTAB surfactant as a mesoporogen [27], while other surfactant-free water-phase routes were mainly applied for dye-adsorption or enzyme-immobilization purposes. Although these reported water-phase protocols can obtain products with MIL-53 topology, most of these materials were only investigated for dye-adsorption performance; their luminescence properties and ratiometric fluorescence-sensing performance toward toxic water-phase anions have not been systematically explored. Our research group has long focused on mild room-temperature aqueous MOF synthesis to avoid harsh heating and toxic solvents. In our previous studies, we successfully constructed water-synthesized Ga-based antibacterial MOFs, Eu-based ratiometric fluorescent probes for Cu^2+^ detection, and Zr–Tb bimetallic luminescent sensors for antibiotic monitoring, fully verifying the reliability and superiority of this green aqueous synthetic strategy [30,31,32]. Nevertheless, few investigations have exploited the luminescence performance of room-temperature water-synthesized NH_2_-MIL-53(Al), especially its unique dual-peak ratiometric fluorescence response toward toxic Cr(VI) in water. Inspired by these preliminary findings, in this work, a luminescent Al-MOF (Al-GM) was rationally designed and mildly synthesized via a facile room-temperature aqueous method using aluminum nitrate as the metal source and 2-aminoterephthalic acid as the organic ligand. The as-prepared Al-GM shows strong blue fluorescence, good stability, and high sensitivity toward Cr_2_O_7_^2−^. With the increase in Cr_2_O_7_^2−^ concentration, the fluorescence intensity of Al-GM is significantly quenched, showing a good linear relationship and low detection limit. Meanwhile, the Al-GM probe exhibits excellent anti-interference ability and fast response kinetics. This work not only provides a mild strategy for the synthesis of Al-GM but also develops a promising fluorescent platform for the rapid and sensitive detection of Cr_2_O_7_^2−^ in environmental water samples.

## 2. Results and Discussion

### 2.1. Characterization of Al-GM

In this work, Al-GM was synthesized via a mild room-temperature aqueous route with aluminum nitrate as the metal source and 2-aminoterephthalic acid (NH_2_-BDC) as the organic ligand. For comparison, traditional NH_2_-MIL-53 was prepared through a solvothermal method using DMF as the reaction solvent. Although Al-GM adopts the NH_2_-MIL-53 topological framework similar to previously reported water-synthesized analogues, distinct synthetic parameters are adopted in our work. We employ aluminum nitrate as metal precursor (instead of aluminum chloride) and avoid any surfactant additives. Even under similar room-temperature aqueous conditions, variations in metal salt anion, pH evolution during reaction, and nucleation kinetics collectively modulate grain size, defect density, and particle stacking behavior, which further give rise to the unique dual-emission luminescence feature of Al-GM that has not been documented in those earlier reports. It should be noted that, although our procedure avoids high-temperature autoclave treatment and harmful organic solvents, a 1 M sodium hydroxide solution is required for ligand dissolution, which should be taken into account when evaluating its green-chemistry merit.

**Figure 1:**
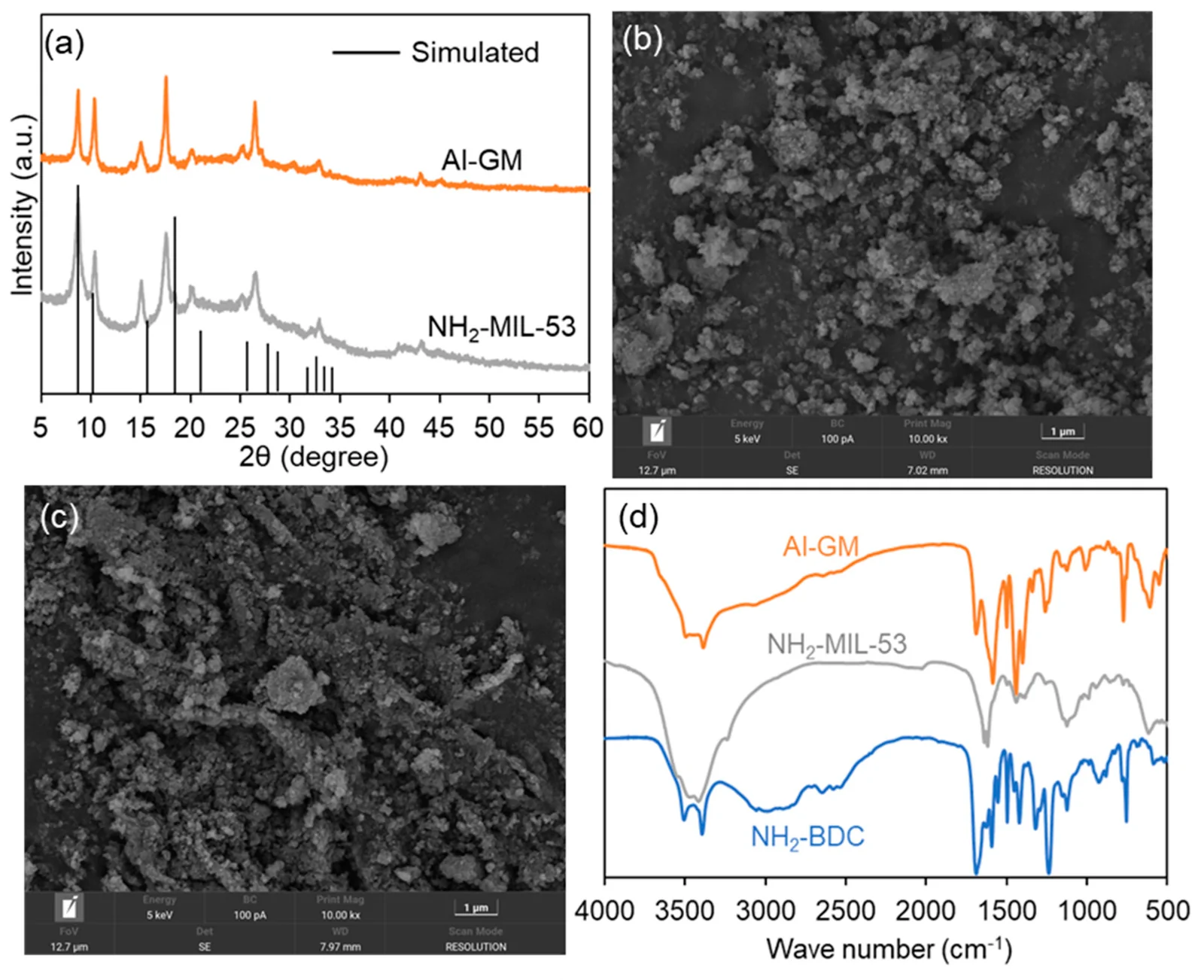
(**a**) XRD patterns of simulated NH_2_-MIL-53, Al-GM, and solvothermally prepared NH_2_-MIL-53. SEM images of (**b**) Al-GM and (**c**) NH_2_-MIL-53. (**d**) FT-IR spectra of NH_2_-BDC, NH_2_-MIL-53, and Al-GM.

X-ray diffraction (XRD) was first employed to compare the crystalline phase and purity of the two as-prepared samples, with the simulated diffraction pattern of NH_2_-MIL-53 as a standard reference (Figure 1a). The characteristic diffraction positions of both samples matched perfectly with the simulated pattern, proving that the two synthetic routes could produce identical NH_2_-MIL-53 crystalline frameworks without introducing miscellaneous crystal impurities. However, obvious distinctions in peak shape and baseline were observed between the two materials. The room-temperature-prepared Al-GM displayed sharper, more symmetric diffraction peaks and a smooth, low-noise baseline, which reflected higher crystallinity and fewer amorphous impurities. In comparison, solvothermal NH_2_-MIL-53 showed relatively widened diffraction peaks and an obviously raised background.

It is worth clarifying that both materials share an identical MIL-53 topological lattice, as evidenced by their consistent diffraction-peak positions. The observed differences in peak width and baseline background could presumably arise from variations in average crystallite domain size, concentration of point-lattice defects, and the number of amorphous impurity phases. For room-temperature aqueous-synthesized Al-GM, larger coherent scattering domains and reduced amorphous by-products may contribute to sharper diffraction signals and a smoother baseline. By contrast, solvothermally synthesized NH2-MIL-53(Al) may contain more local lattice disorder and amorphous impurities, which might account for peak broadening and elevated background noise in its XRD pattern.

Scanning electron microscopy (SEM) was then applied to visually compare the micro-morphological features of the two MOF samples induced by different synthetic conditions. Both samples were assembled from uniform nano-sized primary crystalline grains, which led to an overall similar grain size scale. Nevertheless, obvious differences in particle aggregation modes were observed. The room-temperature-synthesized Al-GM exhibited discrete spherical aggregates formed by loosely packed nanoparticles, with abundant open intergranular voids distributed between isolated clusters (Figure 1b and Figure S1). By contrast, the nanoparticles of solvothermal NH_2_-MIL-53 tended to adhere to one another and extend along a single direction, constructing continuous strip-like stacked frameworks with more compact stacking regions and fewer exposed open gaps (Figure 1c and Figure S2).

Fourier transform infrared (FT-IR) spectroscopy was then used to analyze the coordination interactions and surface functional groups of the free NH_2_-BDC ligand, Al-GM, and NH_2_-MIL-53. The free NH_2_-BDC ligand exhibited an intense band at 1690 cm^−1^ assigned to the C=O stretching of protonated -COOH, which was greatly attenuated in both MOFs, demonstrating that most carboxyl groups of ligands were deprotonated and coordinated with Al^3+^ centers. No obvious absorption signal at 1690 cm^−1^ was detected for solvothermal NH_2_-MIL-53, while an obvious characteristic band around 1630 cm^−1^ existed in the spectra of solvothermal NH_2_-MIL-53 and free organic ligand, whereas no corresponding absorption was found for aqueous Al-GM. In addition, all vibration peaks of aqueous Al-GM in the range of 1000 cm^−1^ to 1500 cm^−1^ are sharp with well-separated sub-peaks, while all absorption bands of solvothermal NH_2_-MIL-53 are severely broadened and overlapped, lacking a fine spectral resolution. These FT-IR distinctions directly demonstrate that synthetic conditions effectively regulate pore impurity content and the structural order of NH_2_-MIL-53 MOFs.

**Figure 2:**
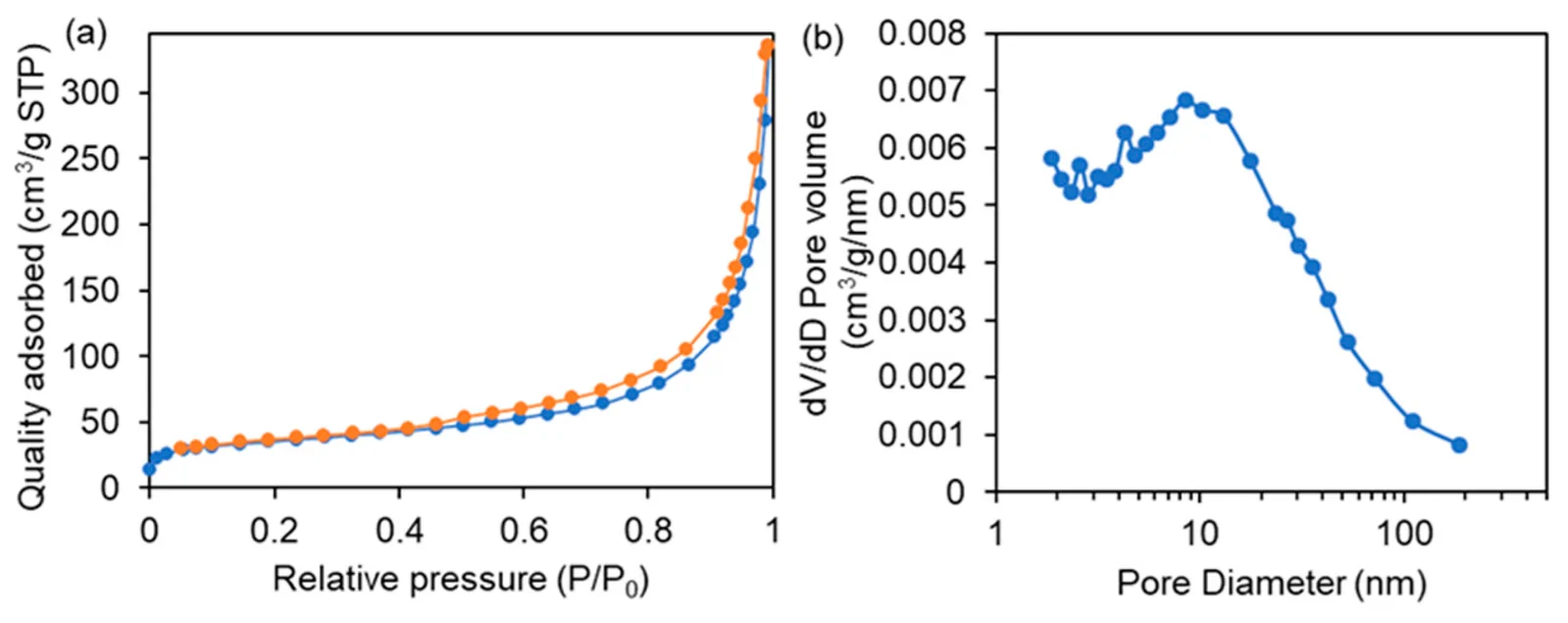
(**a**) N_2_ adsorption–desorption isotherms and (**b**) BJH pore size distribution curve of Al-GM.

N_2_ adsorption–desorption measurement was carried out to characterize the pore structure of Al-GM. As shown in Figure 2a, Al-GM displays a typical type-IV adsorption isotherm with an obvious hysteresis loop at P/P_0_ ranging from 0.4 to 1.0, which is the representative feature of mesoporous materials, confirming that mesopores constitute the main pore structure of Al-GM. At low relative pressure, P/P_0_, the adsorbed nitrogen volume increased moderately, corresponding to monolayer and multilayer adsorption on the material surface. A steep rise in adsorption capacity was observed at a high P/P_0_, which was attributed to the capillary condensation of nitrogen within abundant mesopores. The desorption branch lay below the adsorption branch and formed a closed hysteresis loop, verifying well-interconnected mesoporous channels. The BET specific surface area was calculated as 127.82 m^2^/g. Moreover, the exterior surface area of Al-GM is 102.91 m^2^/g (calculated using the t-plot method), which is higher than the value (79.39 m^2^/g) reported for water-synthesized NH2-MIL-53(Al) [28]. The increased exterior surface area suggests more exposed outer surfaces and accessible amino active sites, which is beneficial for target-analyte contact and subsequent fluorescence-sensing performance. As shown in Figure 2b, the BJH pore size distribution profile shows that the pore diameter of Al-GM spans the range of 2–100 nm, with the maximum pore volume centered at approximately 10 nm. The calculated average pore diameter was 23.18 nm, indicating that mesopores dominated the pore structure with a small fraction of macropores.

**Figure 3:**
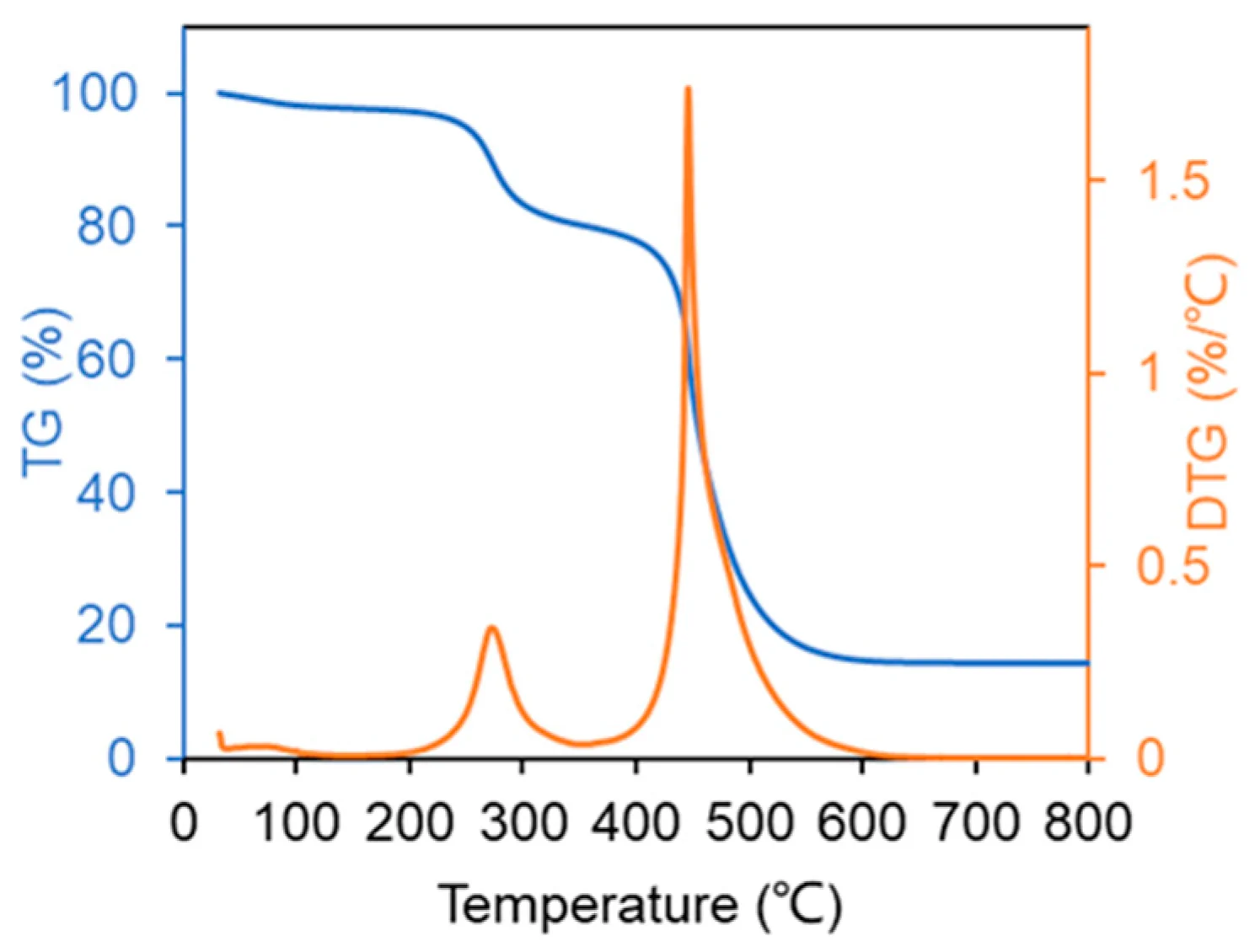
TG-DTG curves of Al-GM.

Thermogravimetric and derivative thermogravimetric (TG-DTG) analyses were carried out from 30 °C to 800 °C under air atmosphere at a heating rate of 10 °C·min^−1^ to investigate the thermal stability of Al-GM. As shown in Figure 3, The blue curve represents the TG mass loss profile, while the orange curve corresponds to the DTG derivative signal. The first mild weight loss occurred from room temperature to 250 °C, accompanied by a weak DTG peak centered at the range of 250–300 °C. This mass reduction was attributed to the evaporation of physically adsorbed water confined within pore channels. The TG curve maintained a flat plateau between 250 °C and 400 °C, revealing that the organic–inorganic MOF skeleton remained structurally intact and exhibited favorable thermal stability at moderate temperatures. A dramatic weight loss was recorded in the range of 400–600 °C, where the TG curve declined steeply and the DTG curve produced an intense sharp peak near 450 °C.

**Figure 4:**
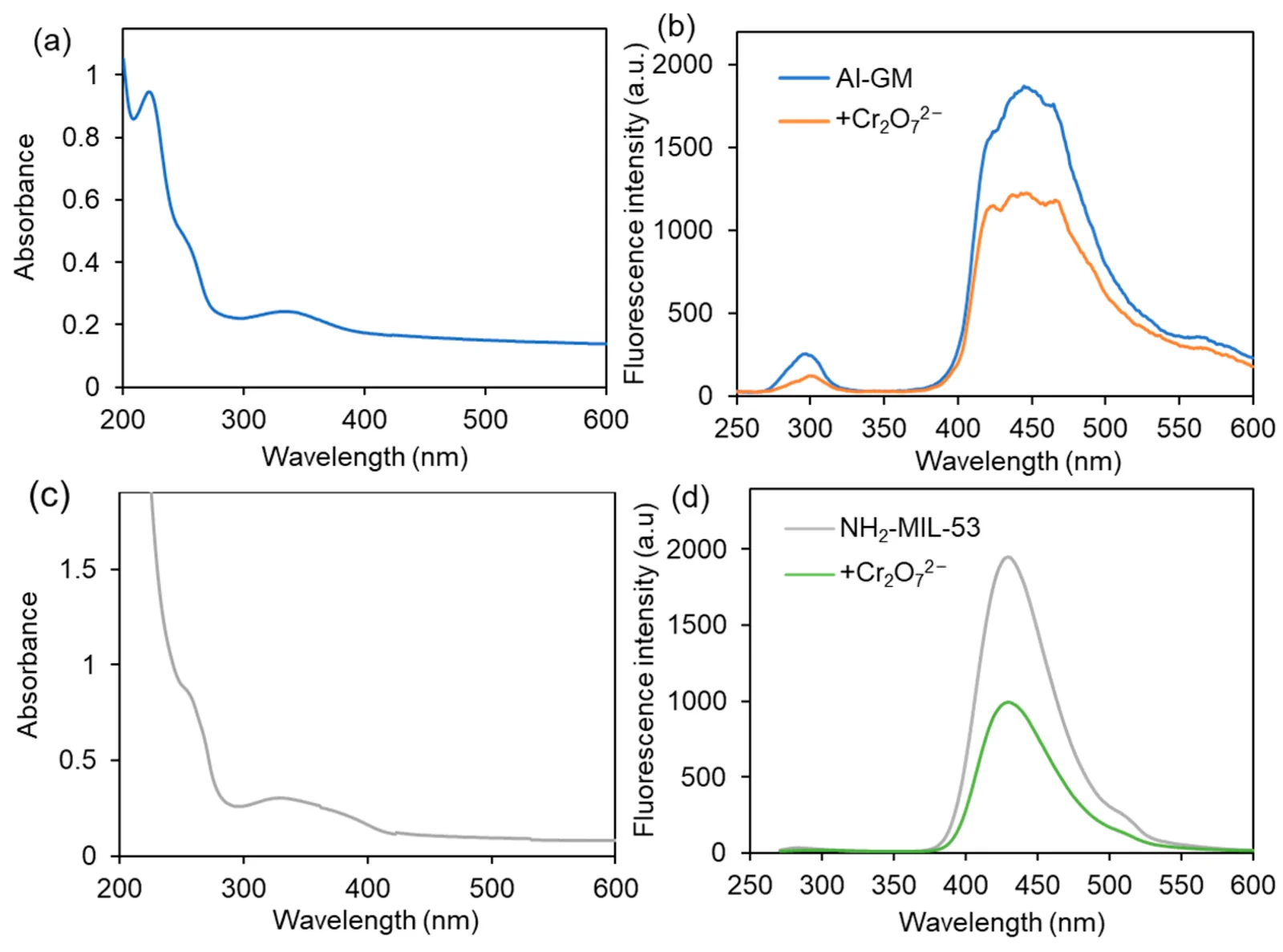
UV–Vis absorption spectra of (**a**) Al-GM and (**c**) NH_2_-MIL-53. Fluorescence spectra of the blank sample and sample treated with 200 μM Cr_2_O_7_^2−^ for (**b**) Al-GM and (**d**) NH_2_-MIL-53.

UV–Vis absorption and fluorescence spectra were recorded to compare the optical properties of Al-GM and NH_2_-MIL-53. As displayed in the UV–Vis absorption curves (Figure 4a,c), both materials exhibit strong absorption signals in the range of 200–300 nm, accompanied by a weak broad absorption band in the range of 300–400 nm. These two absorption bands are likely attributed to the π→π* and n→π* electronic transitions of the organic NH_2_-BDC ligand. Figure 4b,d show the original fluorescence spectra of pristine MOFs (control groups) and the corresponding spectra after the addition of 200 μM dichromate ions. Distinct differences in fluorescence features can be observed between the two samples. Two fluorescence emission signals (i.e., 299 nm and 440 nm) are detected for Al-GM. In contrast, NH_2_-MIL-53 only produces a single emission peak at 440 nm without the characteristic signal near 299 nm. The fluorescence emissions of both materials are probably derived from the intramolecular charge transfer process of the NH_2_-BDC ligand.

Fluorescence quenching occurs for both MOFs upon the introduction of 200 μM Cr_2_O_7_^2−^. For Al-GM, the peaks at 299 nm and 440 nm decrease simultaneously and significantly, which enables the construction of a ratiometric fluorescence-sensing system. By comparison, only the single peak at 440 nm of NH_2_-MIL-53 undergoes quenching, which relies merely on the change in a single signal for detection. The ratiometric sensing strategy can eliminate interferences from external conditions, instrument fluctuations, and sample concentration by calculating the intensity ratio of the two emission peaks, thus greatly improving detection accuracy and anti-interference capability. Accordingly, Al-GM possesses a superior performance for the fluorescence detection of dichromate.

The extra emission band at 299 nm observed for water-synthesized Al-GM can be tentatively ascribed to changes in the local chemical environment of organic ligands originating from different crystallization conditions. Benefiting from higher crystallinity, fewer amorphous impurities, and looser particle stacking in Al-GM, the relative population of distinct excited-state species originating from NH_2_-BDC moieties is modulated. In contrast, solvothermally prepared NH_2_-MIL-53(Al) bears abundant structural defects and exhibits compact grain aggregation. These features open additional non-radiative relaxation pathways and consequently quench the short-wavelength emission near 299 nm. Further spectroscopic characterizations, including fluorescence-lifetime measurements, will be performed in follow-up work to gain a deeper mechanistic understanding of this luminescence difference.

### 2.2. Fluorescence-Sensing Performance

**Figure 5:**
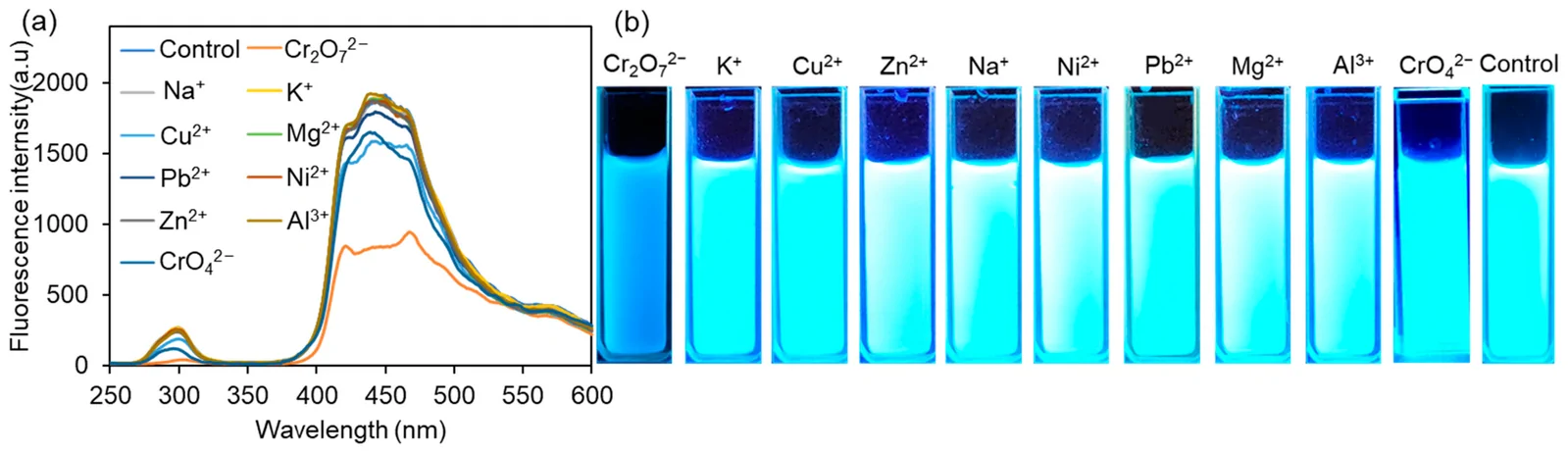
(**a**) Fluorescence spectra of Al-GM after adding various interfering metal ions and Cr_2_O_7_^2−^ at equal concentrations (500 μM); (**b**) corresponding visual fluorescence photographs under UV irradiation.

Numerous coexisting ions (both cations and anions) exist in practical water samples, which are likely to interfere with the fluorescence detection of dichromate. Accordingly, the ion recognition selectivity of the Al-GM probe was investigated, and the results are illustrated in Figure 5. Equivalent concentrations of Na^+^, K^+^, Mg^2+^, Al^3+^, Cu^2+^, Zn^2+^, Ni^2+^, and Pb^2+^ were introduced into Al-GM suspensions using their corresponding nitrate or chloride salts. In addition, CrO_4_^2−^ and Cr_2_O_7_^2−^ were added separately, and the corresponding fluorescence spectra were recorded. As shown in Figure 5a, negligible or moderate fluorescence attenuation can be observed for all the tested metal cations; their accompanying nitrate and chloride counter-anions produce almost no observable interference. CrO_4_^2−^ also induced moderate fluorescence quenching, yet its quenching efficiency was obviously weaker compared with Cr_2_O_7_^2−^. Only the group with dichromate exhibited synchronous and remarkable quenching of both emission peaks. The visual fluorescence photographs further verify this phenomenon: the luminous brightness drastically decreases merely in the Cr_2_O_7_^2−^ group, while the fluorescence intensity of other ion groups remains consistent with the blank control. These findings demonstrate that the Al-GM probe possesses an outstanding specific recognition capability for Cr_2_O_7_^2−^.

**Figure 6:**
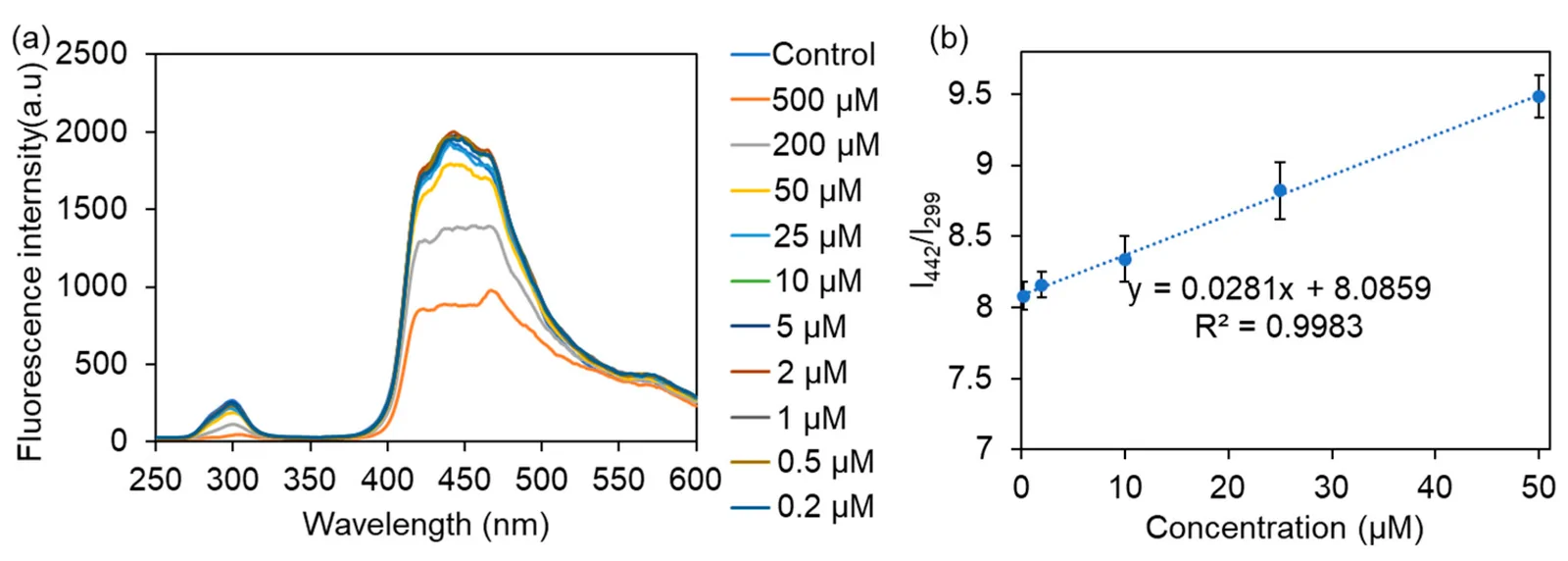
(**a**) Fluorescence spectral evolution of Al-GM with increasing Cr_2_O_7_^2−^ concentration (0.2–500 μM); (**b**) linear fitting plot of intensity ratio I_442_/I_299_ against low-concentration Cr_2_O_7_^2−^.

To evaluate the quantitative detection performance of the Al-GM probe toward Cr_2_O_7_^2−^, a series of dichromate solutions with gradient concentrations were mixed with Al-GM suspensions, and the corresponding fluorescence spectra were recorded as displayed in Figure 6. As the concentration of Cr_2_O_7_^2−^ increased gradually from 0.2 μM to 500 μM, the two characteristic fluorescence peaks of Al-GM at 299 nm and 440 nm declined synchronously and continuously, and the quenching degree showed an obvious dependence on ion concentration.

In the low-concentration range, the intensity ratio of the dual fluorescence peaks exhibited a favorable linear correlation with Cr_2_O_7_^2−^ concentration, enabling the accurate quantitative detection of dichromate via the ratiometric fluorescence signal. Compared with conventional fluorescent probes relying only on single peak intensity, the ratiometric strategy based on dual emission peaks can eliminate detection deviations caused by external factors such as sample concentration, instrument errors, and ambient light, and greatly improve the reliability of quantitative results. Meanwhile, the probe produced an obvious fluorescence response to Cr_2_O_7_^2−^ at a concentration as low as 0.2 μM (0.0432 mg/L), which is far below the commonly stipulated permissible limit of 0.5 mg/L for Cr(VI) in industrial wastewater [33]. When compared with recently reported MOF-based Cr(VI) fluorescent sensors, the LOD values of viologen-based MOF (133 μM) [34], lanthanide–porphyrin MOF (0.2 mg/L, 0.56 μM) [33,35], copper-based MOF (6 μM) [36], and Congo red-modified MOF (8.6 μM) [37] are all inferior to our Al-GM; only a few composite doped MOFs achieve lower limits of detection (0.01 μM, 0.016 μM, and 0.16 μM) [38,39,40]. This demonstrates that Al-GM possesses competitive sensing sensitivity among single-component amino–MOF materials and can meet the requirements of trace detection and discharge monitoring of hexavalent chromium in water. The fluorescence photographs under gradient concentrations also visually revealed that the luminous brightness gradually weakened with the increase in dichromate concentration, which was highly consistent with the spectral results.

**Figure 7:**
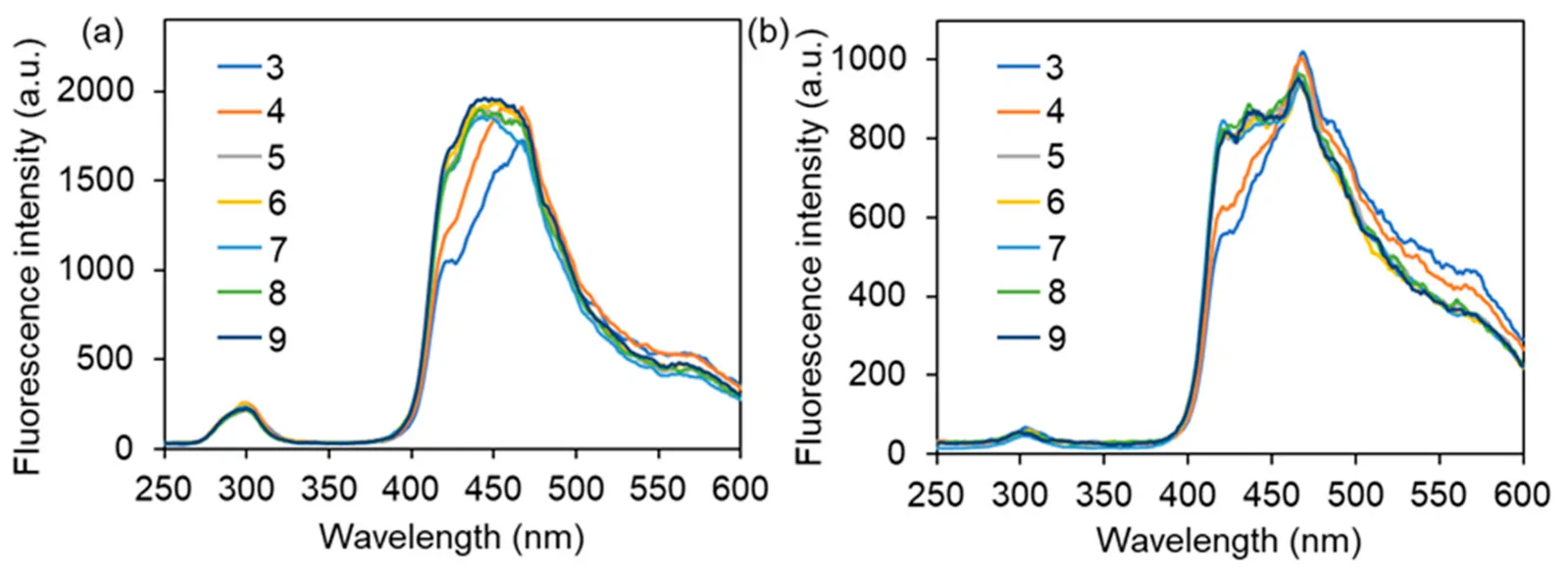
Fluorescence spectra of Al-GM at a pH range of 3–9. (**a**) Al-GM without Cr_2_O_7_^2−^; (**b**) Al-GM after adding Cr_2_O_7_^2−^.

The pH of actual water samples varies widely, and acidity or alkalinity can alter the surface charge of MOFs and the existing form of dichromate ions, further interfering with the recognition performance. Thus, the fluorescence stability and detection capacity of Al-GM were investigated at pH levels ranging from 3 to 9, as shown in Figure 7.

In the absence of Cr_2_O_7_^2−^, the dual-peak fluorescence intensity of Al-GM remained nearly stable at a pH range of 5–9. When the solution pH dropped to 3 and 4 under strongly acidic conditions, the fluorescence intensity decreased obviously, especially at pH = 3 with a prominent attenuation. This phenomenon could be ascribed to the partial destruction of the MOF coordination framework by high-concentration H^+^, and the protonation of amino groups altered the electronic distribution of the skeleton, which greatly aggravated non-radiative energy loss.

After adding equivalent Cr_2_O_7_^2−^ into solutions with different pH values, synchronous dual-peak quenching could be steadily achieved at a pH range of 5–9 with minor differences in fluorescence response. However, the quenching efficiency was remarkably weakened under strongly acidic conditions (pH = 3, 4), accompanied by an obvious decline in sensing sensitivity. Overall, Al-GM can reliably identify Cr_2_O_7_^2−^ in neutral and weakly acidic/alkaline water, showing promising application prospects for conventional natural water and industrial wastewater monitoring.

### 2.3. Possible Sensing Mechanism

**Figure 8:**
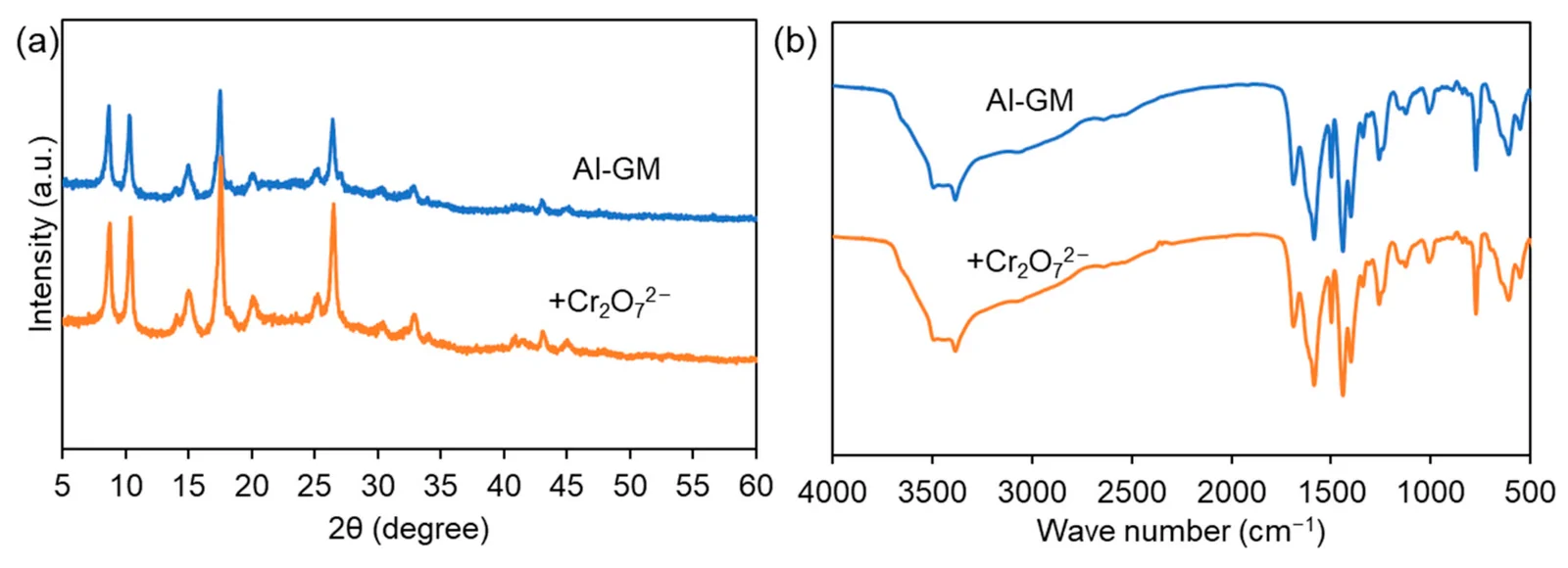
(**a**) XRD patterns of Al-GM and Cr_2_O_7_^2−^-adsorbed Al-GM; (**b**) FT-IR spectra of Al-GM and Al-GM treated with the Cr_2_O_7_^2−^ solution.

To clarify the intrinsic fluorescence-sensing mechanism, XRD and FT-IR spectra of Al-GM before and after interaction with Cr_2_O_7_^2−^ were compared. As displayed in Figure 8a, all characteristic diffraction peaks of samples before and after dichromate adsorption are well-matched, without new diffraction signals, peak disappearance, obvious shifts, or broadening. This result confirms that the crystal framework of Al-GM can remain intact during the recognition process. Cr_2_O_7_^2−^ merely diffuses into the pore channels and interacts weakly with framework sites on the surface or inside pores, rather than breaking coordination bonds to form new crystalline phases.

All characteristic vibration bands of the organic ligand are fully retained in FTIR spectra before and after adsorption (Figure 8b), demonstrating the integrity of the organic coordination skeleton. Only negligible changes in absorption intensity are observed for the NH_2_ deformation vibrations (1500–1600 cm^−1^) and N-H stretching band (3300–3500 cm^−1^). No obvious peak shift or new characteristic peaks are detected, which indirectly verifies that amino sites on the framework participate in weak interactions with Cr_2_O_7_^2−^.

The amino groups anchored on ligands tend to carry positive charges in aqueous media, which can bind negatively charged Cr_2_O_7_^2−^ via electrostatic attraction. Meanwhile, intermolecular hydrogen bonds are likely to form between N-H groups of amino sites and oxygen atoms of dichromate. The synergy of these two weak interactions enables specific capture of Cr_2_O_7_^2−^, while other coexisting metal cations cannot form such matched interactions, which explains the outstanding ion selectivity of the Al-GM probe.

## 3. Materials and Methods

### 3.1. Reagents and Chemicals

Aluminum nitrate hydrate (Al(NO_3_)_3_·9H_2_O), 2-aminoterephthalic acid (NH_2_-BDC), sodium chloride, potassium chloride, magnesium chloride, copper chloride, lead nitrate, nickel chloride, and potassium dichromate were of analytical grade and bought from Macklin Biochemical Co., Ltd. (Shanghai, China). The water used in this work was ultrapure water.

### 3.2. Synthesis of Al-GM and NH_2_-MIL-53

**Figure 9:**
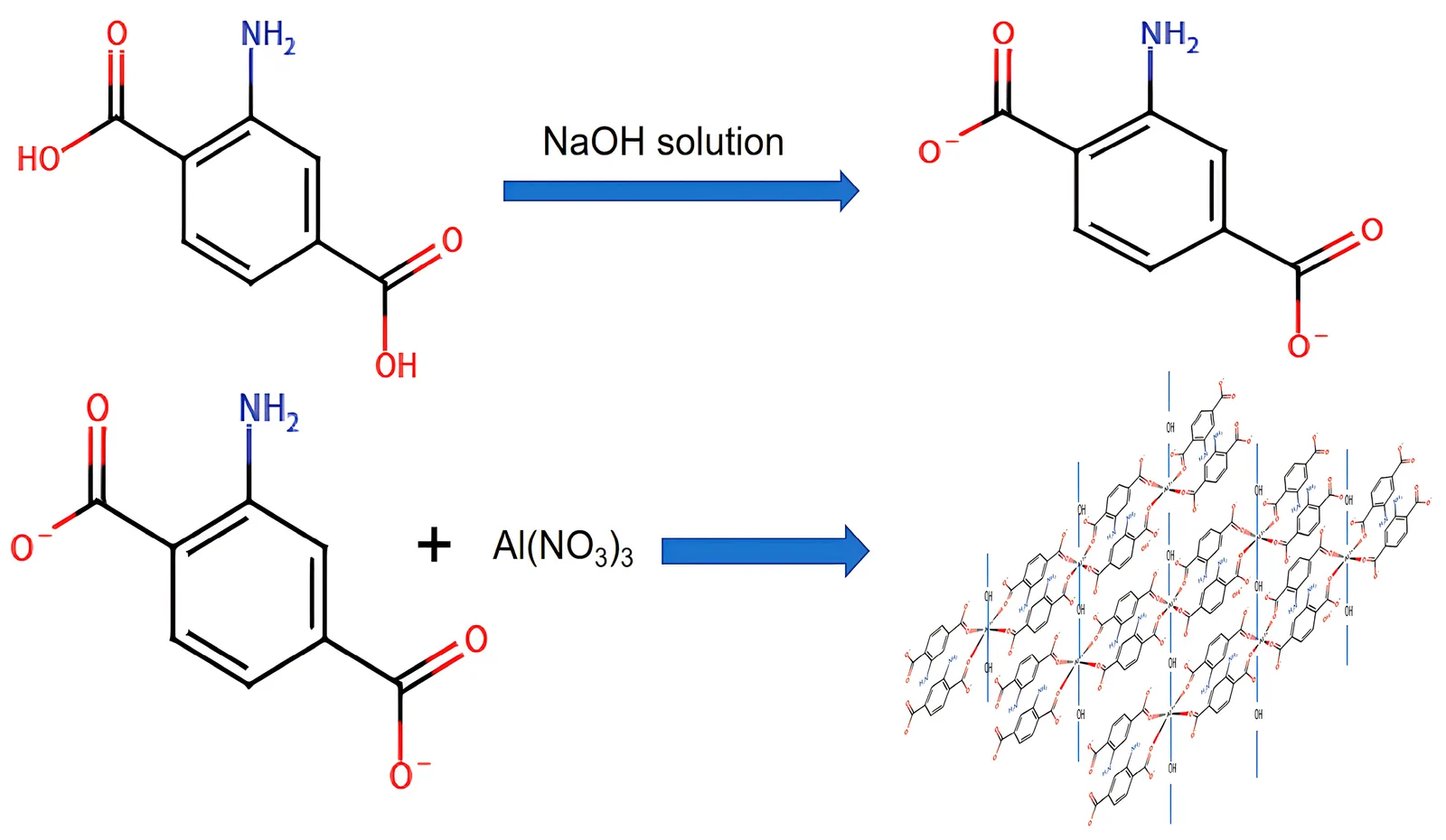
Schematic illustration for the room-temperature aqueous assembly process of Al-GM. The NH_2_-BDC ligand undergoes deprotonation in the NaOH aqueous solution, followed by coordination with Al^3+^ ions from aluminum nitrate to form Al-GM with a MIL-53-type topology. The right-bottom panel shows a simplified schematic representation of the assembled layered-framework structure.

The Al-GM sample was obtained via a room-temperature aqueous-phase coordination reaction, and the assembly process is schematically illustrated in Figure 9. Specifically, 125 mg of aluminum nitrate hydrate was dissolved in 10 mL of deionized water to obtain the solution A. Meanwhile, 64 mg of NH_2_-BDC was added in another 10 mL of deionized water, and dissolved by addition of NaOH (1 M), which is denoted as solution B. Subsequently, solution A was slowly added dropwise into solution B under continuous magnetic stirring. After the completion of dropping, the mixed system was stirred further for 1 h to ensure a sufficient reaction. The resulting yellow precipitate was collected by centrifugation at 14,800 rpm for 5 min, washed twice with deionized water, and centrifuged again for subsequent collection. The obtained samples were dispersed in water and stored at 4 °C for further experiments. The final solid products were obtained by vacuum drying (60 °C, 12 h).

For the synthesis of NH_2_-MIL-53, 125 mg of aluminum nitrate hydrate and 64 mg of NH_2_-BDC were dissolved in DMF (10 mL). The mixture was transferred into a Teflon-lined stainless-steel autoclave and subjected to thermal treatment at 120 °C for 12 h. Once the heating program finished, the reactor was naturally cooled down to ambient temperature. The resultant mixture was separated via centrifugation, and the supernatant liquid was discarded. The sediment was rinsed twice with deionized water repeatedly, and the purified solid precipitate was collected and dried under vacuum conditions (60 °C, 12 h).

### 3.3. Characterization

A scanning electron microscope (SEM, ZEISS Sigma 360, Oberkochen, Germany) was used to characterize the morphological features. Fourier transform infrared spectroscopy (FT-IR, ThermoFisher Scientific, Nicolet iS50, Waltham, MA, USA) was employed to identify surface functional groups using the KBr pellet method. X-ray diffraction (XRD, Rigaku SmartLab, Tokyo, Japan) was utilized to analyze the crystalline structure of the samples. Fluorescence spectra were tested using an F97 fluorescence spectrophotometer (Shanghai Lengguang Technology, Shanghai, China). The pore structure and specific surface area were determined using a surface area and porosity analyzer (BET, Micromeritics ASAP 2460, Norcross, GA, USA). Thermogravimetric analysis (TGA) was carried out using a thermal analyzer (HITACHI STA200, Tokyo, Japan) in air atmosphere with a heating rate of 10 °C·min^−1^ from room temperature to 800 °C.

### 3.4. Fluorescent Detection of Cr_2_O_7_^2−^

The aqueous solution of Al-GM (0.2 mg/mL) was prepared by dispersing Al-GM into ultrapure water under ultrasound for 10 min. Then, 1 mL of Cr_2_O_7_^2−^ solution at various concentrations was added to 1 mL of Al-GM solution. After incubation for 1 min, fluorescence emission spectra were recorded. All experiments were performed at room temperature and repeated at least three times for reliability.

## 4. Conclusions

In conclusion, amino-functionalized Al-based MOF (Al-GM) was prepared via room-temperature aqueous synthesis and employed as a ratiometric fluorescent probe for Cr_2_O_7_^2−^ detection in water. Structural characterizations confirm that the material possesses high crystallinity and abundant mesopores with abundant exposed amino recognition sites, enabling dual-channel fluorescence emission signals. Performance tests reveal that the probe exhibits prominent selectivity toward Cr_2_O_7_^2−^ with strong anti-interference capacity, a wide linear detection range and low limit of detection, and stable sensing performance within the pH range of conventional water samples. XRD and FTIR spectra before and after dichromate adsorption verify that the MOF framework remains intact during detection, and Cr_2_O_7_^2−^ is selectively captured by amino sites via the combined effect of electrostatic interaction and hydrogen bonding. This mild synthetic route provides a low-cost, practical fluorescent sensor, offering a new strategy for the rapid detection of trace Cr(VI) in aquatic environments.

## Data Availability

The data presented in this study are available on request from the corresponding authors.

## Supplementary Materials

The following supporting information can be downloaded at: https://www.mdpi.com/article/10.3390/molecules31173051/s1, Figure S1: SEM images of Al-GM. Figure S2: SEM images of NH_2_-MIL-53.
