# Supplementary material for: Room-Temperature Aqueous Synthesis of Amino-Functionalized Al-Based MOF as a Ratiometric Fluorescent Probe for Aqueous Dichromate Detection
Source: Molecules. 2026 Aug 31;31(17):3051. doi: 10.3390/molecules31173051 (PMC13567672; doi:10.3390/molecules31173051)
Supplement: Supplementary file 1 [file molecules-31-03051-s001.zip › molecules-4510401-supplementary.pdf]

Room-Temperature Aqueous Synthesis of Ami-no-Functionalized Al-Based MOF as Ratiometric Fluorescent Probe for Aqueous Dichromate Detection

Chuyao Huang [1,#], Yanxiu Zhang [1,#], Shu Li [2], Rui Lin [1], Yunfan Zhang [1], Jingqi Chen [1], Yutong Sun [1], Yue Wang [1] and Shuo Liu [1,*]

[1] School of Energy and Chemical Engineering, Tianjin Renai College, Tianjin 301636, China;

[2] Technology Center of Shenyang Customs, Shenyang 110016, China; linda_0915@126.com

[#] The authors contributed equally to this work.

* Correspondence: liushuo@nankai.edu.cn, liushuo@tjrac.edu.cn.

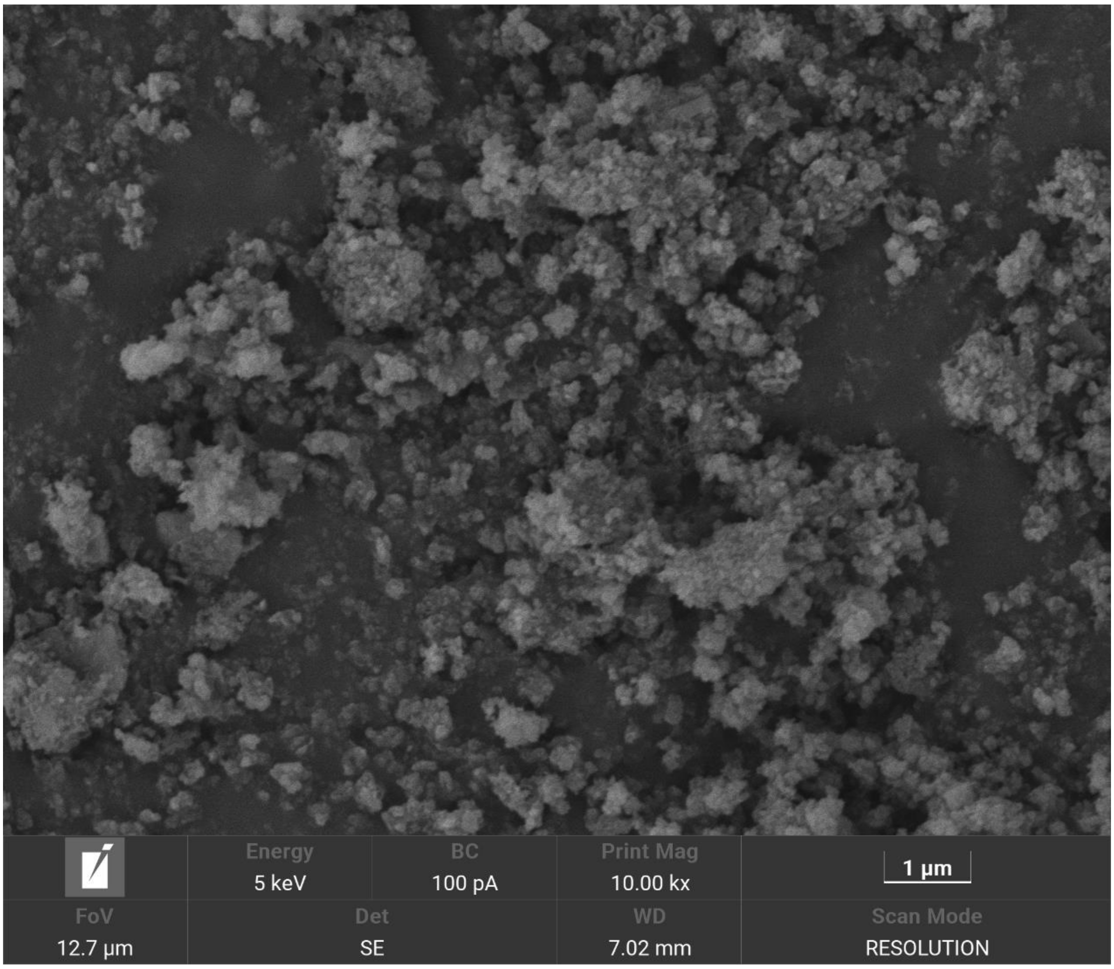

Figure. S1 SEM images of Al-GM.

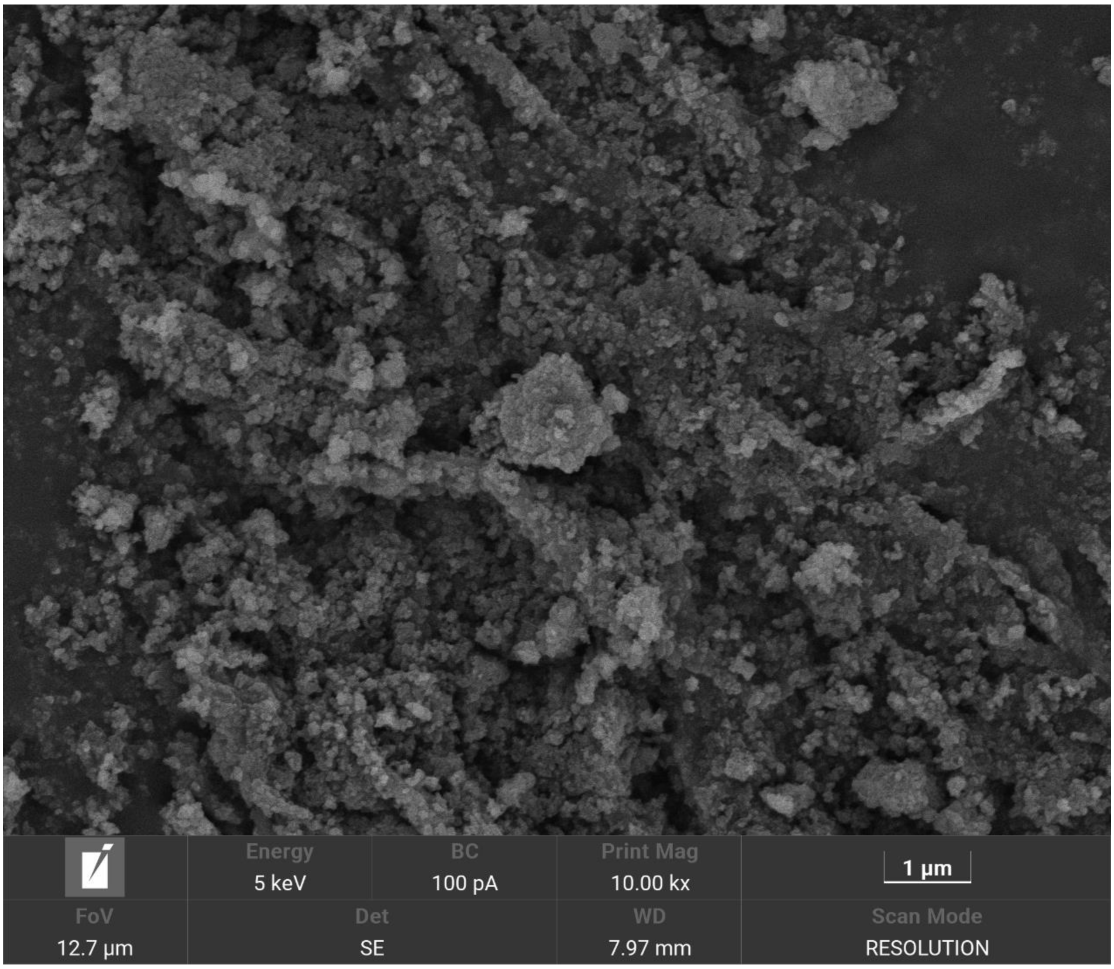

Figure. S2 SEM images of NH_2-MIL-53.
